# Sirolimus on Gorham-Stout disease. Case report.

**Published:** 2016-12-30

**Authors:** Vanessa García, Gloria Alonso-Claudio, M-Teresa Gómez-Hernández, Antonio-J Chamorro

**Affiliations:** 1 Departamento de Medicina Interna. Hospital Clínico Universitario de Salamanca. Salamanca. España.; 2 Departamento de Cirugía Torácica. Hospital Clínico Universitario de Salamanca. Salamanca. España

**Keywords:** Pleural effusion, osteolysis essential, metrorrhagia, sirolimus, Gorham-Stout disease, prognosis, rare diseases, cell proliferation.

## Abstract

**Background:**

Gorham-Stout disease (GSD) is a rare disease of unknown etiology characterized by vascular proliferation that produces destruction of bone matrix.

**Case description:**

This case is about 43 year old woman who begins with pain in sternum, dyspnea, abdominal mass and, serous-hematic pleural effusion. Imaging tests were performed showing lesions on 6^th^ and 10^th^ left ribs archs. Later, a thoracotomy was performed observed absence of the end of the 6^th^ and lung, pleural and costal biopsy was token. The histologic features described lymphatic vascular proliferation in bone tissue of chest wall. Other pathologies were excluded and in view of the findings, GSD diagnosis was made.

**Treatment and outcome:**

treatment was initiated with sirolimus achieving remission of the disease after the first month; however, because the presence of metrorrhagia the treatment was discontinued, reappearing symptoms afterwards. For that reason the treatment was restarted getting disappearance of the symptoms again, 4 weeks later.

**Clinical relevance:**

we present the first clinical cases of EGS with pleural effusion with response to sirolimus treatment that could be an alternative to the current therapy.

## Resumen

**Antecedentes:**

La enfermedad de Gorham-Stout (EGS), es una enfermedad poco común, de etiología desconocida, caracterizada por la proliferación vascular que produce destrucción de la matriz ósea.

**Caso clínico:**

Se presenta el caso de mujer de 43 años que comienza con dolor en el esternón, disnea y tumoración abdominal junto con derrame pleural izquierdo de características serohemáticas como forma de presentación de una EGS. En pruebas de imagen que mostraron lesiones líticas en el 6º y 10º arcos costales izquierdos. Posteriormente se realizó toracotomía con biopsia pulmonar, pleural y costal observándose ausencia del extremo de la 6ª costilla. En el estudio histopatológico se describe proliferación vascular linfática en tejido óseo de pared costal. Se excluyeron otras patologías y se diagnosticó EGS.

**Tratamiento y resultado:**

Se inició tratamiento con sirolimus consiguiendo remisión completa desde el primer mes. Sin embargo, tras la suspensión del tratamiento por metrorragias, presentó reaparición de los síntomas. Se decide entonces reiniciar el tratamiento, consiguiendo nuevamente desaparición de los síntomas, tras 4 semanas de tratamiento.

**Relevancia clínica:**

Se presenta el primer caso clínico de EGS en edad adulta con derrame pleural asociado y con respuesta clínica a sirolimus, fármaco que podría ser una alternativa a la terapéutica actual.

## Introduction

Gorham-Stout disease (GSD), or essential osteolysis, is a rare disease characterized by the proliferation of vascular channels which leads to the destruction and resorption of bone mass. It has an unknown origin ^1^.

The first case published in the literature of "vanishing bone disease", as this condition is also known, was described by Jackson in 1838. In his study, the author described the progressive disappearance of a humerus ^2^. In 1955, Gorham *et al*., published a series with 24 cases of their own and other cases with similar clinical and histological characteristics ^3^. There are approximately 185 described cases with no differences regarding sex or a clear pattern of hereditary transmission. However, there is a predominant incidence of the disease during the first decades of life ^1^. The most common initial symptom is the finding of pathological fractures, but these cases are generally unspecific ^4^, and they can affect any part of the skeleton, although with a preference for flat bones, such as the jaw, ribs, shoulder and pelvis. Its histopathology is characterized by proliferation of small vessels or by lymph channels being replaced by benign fibrovascular tissue ^1^. It generally has a good prognosis, although pulmonary complications secondary to lesions of the thoracic skeleton accompanied by chylothorax and vertebral compromise are rare but fatal complications for this condition ^5^.

There is no clear consensus on the most adequate treatment. A medical approach (interferon alfa-2b and bisphosphonates are some of the most common choices), surgery to reduce the flow towards the pleural cavity (ligation of the thoracic duct, pleurodesis and pleurectomy), radiotherapy and chemotherapy (mainly bleomycin and cyclophosphamide) are options which have been tried in isolated cases with differing degrees of success ^1^.

We present a case of GSD complicated with pleural effusion in which clinical and radiological remission was achieved with a single lead dose of sirolimus 4 mg and maintenance doses of 1.5 mg/day afterwards.

## Case report

The patient is a 43-year-old woman who was admitted with thoracic pain, dyspnea on slight exertion and tumor formation in the left hypochondrium. In her personal history she showed recurrent dislocations of the right shoulder and endometriosis which had been diagnosed two years before. One year before admission, she started showing mechanical pain on the sternal region together with inflammatory signs, without previous trauma. Six months prior to her admission, she presented progressive mechanical pain on the left side, together with formation of a tumor in the left hypochondrium which slowly increased in size, and progressive dyspnea which became dyspnea on slight exertion without hemoptysis, fever, feeling of dysthermia, chills, rigors or any other infectious or respiratory symptoms. She did not report weight loss, asthenia or anorexia. The examination revealed pain and thickening of the 10^th^ left posterior rib arch, pain on palpation on the lower third portion of the sternal region, and diffuse tumor in the left hypochondrium with surface hematoma which was poorly delimited, soft and painless. On cardiopulmonary auscultation, the patient showed semiology of pleural effusion in the lower and middle regions of the left hemithorax. The analysis on admission revealed normal CBC and biochemistry values without elevation of acute-phase reactants. Table 1 shows the analytical data on admission:

**Table 1:** Analysis on admission of patient.

Parameter	Value
Glucose	98 mg/dL
Urea	32 mg/dL
Creatinine	0.63 mg/dL
Total bilirubin	0.56 mg/dL
Proteins	7.5 g/dL
Albumin	4.7 g/dL
Hemoglobin	11.9 gr/dL
Fibrinogen	400 mg/dL
C-reactive protein	1.50 mg/dL
Osmolarity	267 mOsmol/kg.
Aspartate Transaminase (AST)	20 UI/L
Alanine Transaminase (ALT)	17 UI/L
Alkaline phosphatase (ALP)	63 UI/L
Gamma-glutamyltransferase (GGT)	21 UI/L
Lactate dehydrogenase (LDH)	157 UI/L
Erythrocyte sedimentation rate (ESR)	27 mm
Prothrombin time	98%
Hematocrit	34.5%
Leukocytes	6.38 x 10³/μL
Platelets	456 x 10³/μL
Activated Partial Thromboplastin Time	29.5 s
INR	1.01

**Figure 1.**
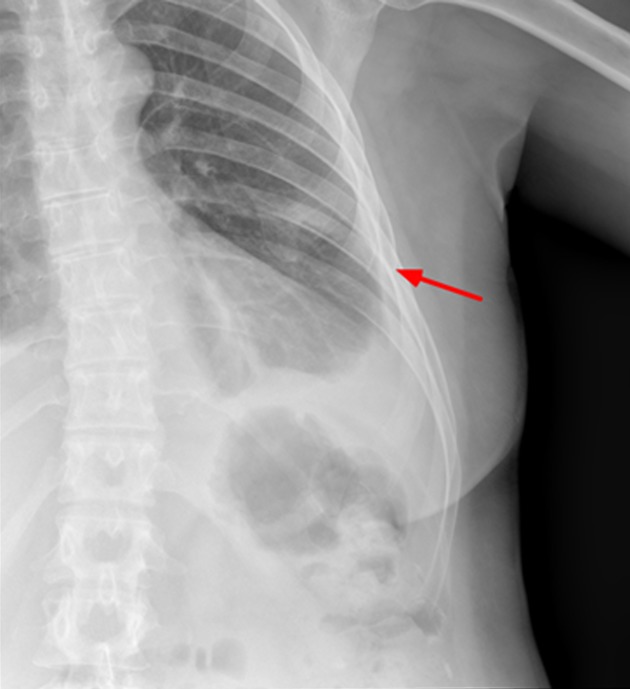
X-ray of left rib cage which may show partial disappearance of the 6^th^ left lateral and anterior rib arch.

The thoracic X-ray reveals pleural effusion which occupies two thirds of the left hemithorax and the left rib cage. Partial absence of the 6th left costal arch may be appreciated as well (Fig. 1).

**Figure 2.**
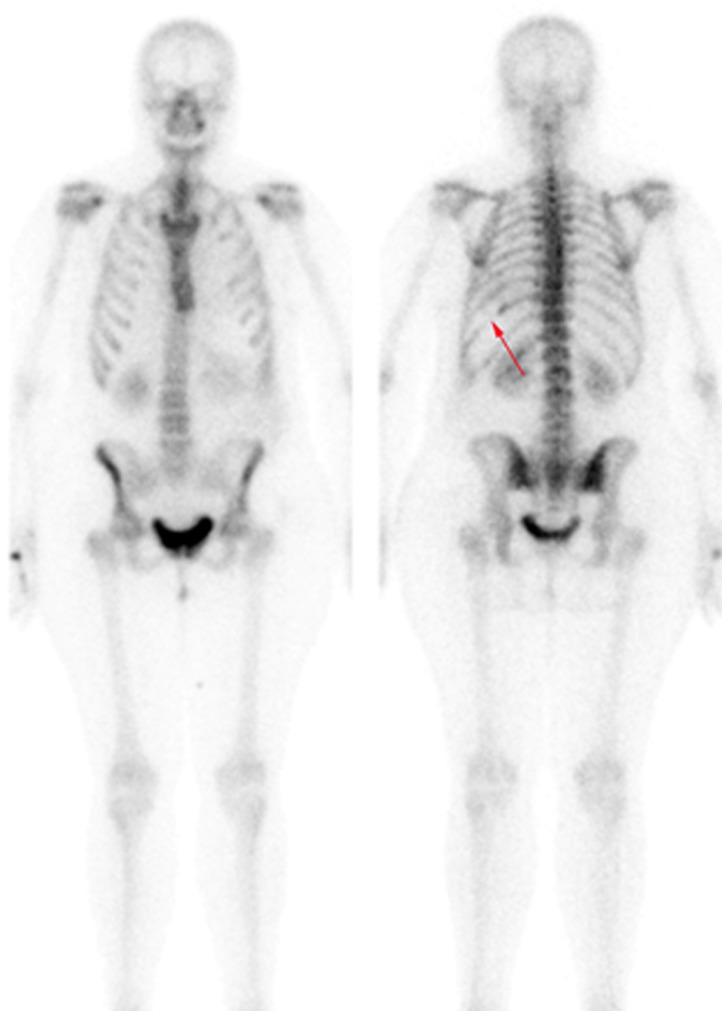
Bone scintigraphy reveals partial absence of the 10^th^ left posterior rib arch.

**Figure 3.**
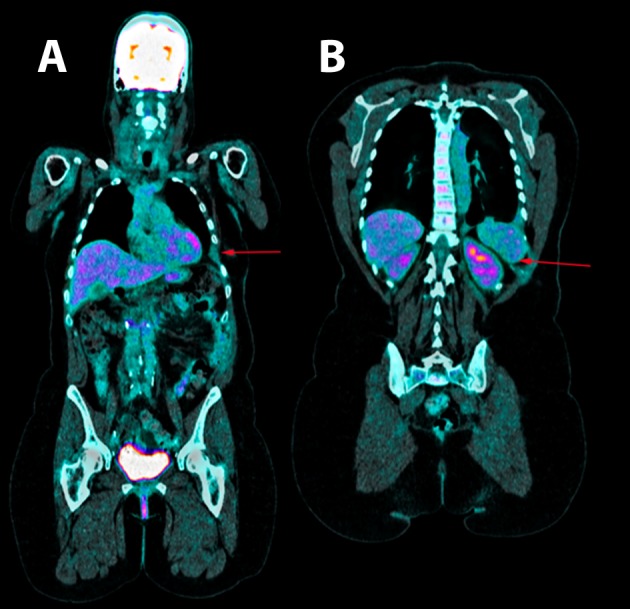
Positron emission tomography. A. shown the absence of the 6^th^ and B. 10^th^ left ribs arches.

Thoracentesis was performed for evacuation with output of serosanguineous fluid with the following analytical data: glucose 97 mg/dl, LDH 179 UI/l, erythrocytes 571100/ μL, leukocytes 11620/ μL (3% polymorphonuclear, 97% mononuclear), proteins 5.3 g/dl, pH 7.43, rheumatoid factor 9.1 UI/ml, amylase 27 UI/l, cholesterol 86 mg/dl, triglycerides 26 mg/dl, adenosine deaminase (ADA) 13 UI/l. Negative flow cytometry of the pleural fluid for lymphoid infiltration. Cytology of the fluid without cellular atypias. Cultures of the pleural fluid for bacteria, mycobacteria, parasites and fungi were negative. Also, the Mantoux test and the gamma interferon test with quantiferon-TB(r) were negative for latent tuberculosis infection. The serology tests for hepatotropic viruses and human immunodeficiency virus (HIV) were negative. The mammography and the gynecologic ultrasound were normal. Both the bone scintigraphy (Fig. 2) and the CT scan (Fig 3), thoracic MRI and PET revealed hypoplasia and an osteolytic lesion in the 10^th^ and 6^th^ left rib arches together with effusion, significant left pleural inflammation and involvement of adjacent soft parts.

A diagnostic thoracotomy was performed, and it showed important signs of inflammation and pleural hyperemia without signs of malignancy or granulomas. Samples of bone, pleura and pulmonary parenchyma were obtained. The histological results of the pleural and pulmonary tissue showed findings compatible with an unspecific inflammatory process. The rib biopsy revealed a chronic inflammatory process with mainly plasmacytoid infiltrate and granulation tissue with many artifacts due to decalcification. In view of these findings and the possibility of actinomycosis, empiric therapy was started with penicillin (doses of 6 million UI/6h for 4 weeks), pending the results of the polymerase chain reaction for *Actinomices sp*. Given the lack of improvement and the negative result of the PCR test, a new empiric therapy with prednisone was started at a dose of 1 mg/kg/day. In spite of these treatments and the placement of a pleural drain, the patient presented recurring pleural effusion, which in the last occasion was bilateral, with right predominance and larger abdominal tumor formation. The vascular endothelial growth factor (VEGF) was measured and it showed elevated levels (287 pg/mL, normal range 0-128.9 pg/mL). At this stage, the case was reviewed again in view of the clinical, analytical and radiological findings, and although the initial histological results were not compatible, a diagnosis of GSD was made and it was decided to start treatment with sirolimus after the patient signed her informed consent. The therapy was administered initially with a single dose of 4 mg (lead dose), and afterwards at a rate of 1.5 mg/day. The response was very satisfactory from the beginning, with disappearance of the symptoms of dyspnea, pain and abdominal tumor formation. The treatment was maintained over 10 months, with sirolimus levels slightly below the limits established by the laboratory (3.8-4.3 ng/mL; threshold: 4 ng/mL minimum and 10 ng/mL maximum), which were monitored every two months. However, due to the suspicion of a relationship between the administration of sirolimus and episodes of abundant metrorrhagia, the treatment was suspended. A few days later, dyspnea, rib pain and particularly the abdominal tumor formation reappeared. The treatment with sirolimus was restarted, with completer remission at the 4^th^ week and normal menstruation. Then, after a reassessment of the biopsy and particularly of the bone samples from the rib arch with immunohistochemical techniques, areas of lymphatic vascular proliferation were observed with D2-40 monoclonal antibody (useful as a specific marker of lymphatic vessels), compatible with the diagnosis of GSD. Since then, and two years after the onset of the symptoms and the analytical and radiological monitoring, the patient is asymptomatic, without changes in the complementary tests.

## Discussion

In GSD, massive and progressive osteolysis is caused by the abnormal proliferation of endothelial capillaries with vascular or lymphatic origin. Although the mechanism of resorption is not known, some researchers have found an increased activity of osteoclasts ^6^ ^,^ ^7^ together with an associated elevation in VEGF and, in other cases, with increased levels of interleukin 6. In our case, we observed elevated VEGF levels which might be used during monitoring and which correlated with the symptoms of the patient. GSD is considered a diagnosis of exclusion, with clinical, radiological and histopathological findings in absence of a hereditary, metabolic, neoplastic, immunological or infectious cause, and in most cases there are no laboratory findings ^8^. From a radiological perspective, the first changes are similar to those found in osteoporosis. A MR image shows disappearance of the bone morphology and areas with increased or reduced enhancement, which may represent different stages of hemorrhage ^9^. In our patient, a diagnosis was reached through clinical and radiological findings with images compatible with lytic lesions, after a reasonable elimination of other pathologies with a neoplastic, infectious, inflammatory or metabolic origin, and also thanks to compatible histopathological findings. Although there are some diagnostic criteria ^10^, they are very rarely used by the authors in the literature for diagnosis, and many of them only use them to reach a diagnosis of exclusion in an adequate clinical context ^1^ ^,^ ^11^ ^,^ ^12^.

Some of the main GSD complications are chylothorax and, less commonly, serosanguineous pleural effusion. These complications are associated with an increase in mortality ^13^. 

There are surgical, radiotherapeutic and medical treatments available. This last category includes mainly octreotide, bevacizumab, propranolol, interferon alfa-2b and bisphosphonates (these two last drugs are the most commonly used ones, either independently or combined). They all show disparate individual results, and for this reason none of them has been established as a standard treatment given the lack of studies on this pathology. Sirolimus is indicated to prevent rejection in transplanted patients, and its mechanism of action inhibits the FK506-binding protein 12-rapamycin-associated protein 1 (FRAP1), which causes a decrease in protein synthesis and, consequently, in cellular proliferation and angiogenesis ^14^. In this regard, a therapeutic alternative has been essayed recently with sirolimus in pediatric patients with vascular and lymphatic anomalies, including GSD, due to its antiangiogenic activity, with excellent results ^14^. Also, a clinical essay is currently being carried out with this group of patients, with significant preliminary results. For this reason, it was prescribed in our case ^1^ ^,^ ^15^. It is worth pointing out that sirolimus was administered at low doses with good clinical response, in spite of the fact that sometimes the registered levels were below the minimum threshold suggested by the laboratory (4 ng/mL), unlike the other two cases published in the literature, one with pleural effusion (with levels around 9-12 mcg/L) ^16^ and the other with bone involvement exclusively (10-15 ng/mL) ^17^. Some of the most common secondary effects are: infections (fungal, viral, bacterial or caused by Herpes simplex), thrombocytopenia, anemia, leukopenia, hypocalcemia, hypophosphatemia, diabetes, hypertension, menstrual disorders, liver and kidney alterations, and increased risk of cancer, mainly lymphoma and skin cancer ^14^ ^,^ ^15^. In our case, the patient showed metrorrhagia, a symptom which did not reappear when the treatment was reintroduced. To date, the patient has not presented any other secondary effect.

Finally, the recurrence of symptoms after the treatment was suspended and the clinical response after the reintroduction of sirolimus reveal a significant role in the control of GSD in this particular case.
